# Molecular Epidemiology of SARS-associated Coronavirus, Beijing

**DOI:** 10.3201/eid1109.040773

**Published:** 2005-09

**Authors:** Wei Liu, Fang Tang, Arnaud Fontanet, Lin Zhan, Tian-Bao Wang, Pan-He Zhang, Yi-He Luan, Chao-Yang Cao, Qiu-Min Zhao, Xiao-Ming Wu, Zhong-Tao Xin, Shu-Qing Zuo, Laurence Baril, Astrid Vabret, Yi-Ming Shao, Hong Yang, Wu-Chun Cao

**Affiliations:** * Beijing Institute of Microbiology and Epidemiology, Beijing, People's Republic of China;; † Institut Pasteur, Paris, France;; ‡ Beijing Armed Force Hospital, Beijing, People's Republic of China;; § Beijing Institute of Basic Medical Sciences, Beijing, People's Republic of China;; ¶ Caen University, Caen, France;; # Chinese Center for Disease Control and Prevention, Beijing, People's Republic of China

**Keywords:** severe acute respiratory syndrome, molecular epidemiology, single nucleotide variation, RT-PCR, research

## Abstract

Single nucleotide variations (SNVs) at 5 loci (17564, 21721, 22222, 23823, and 27827) were used to define the molecular epidemiologic characteristics of severe acute respiratory syndrome–associated coronavirus (SARS-CoV) from Beijing patients. Five fragments targeted at the SNV loci were amplified directly from clinical samples by using reverse transcription–polymerase chain reaction (RT-PCR), before sequencing the amplified products. Analyses of 45 sequences obtained from 29 patients showed that the GGCTC motif dominated among samples collected from March to early April 2003; the TGTTT motif predominanted afterwards. The switch from GGCTC to TGTTT was observed among patients belonging to the same cluster, which ruled out the possibility of the coincidental superposition of 2 epidemics running in parallel in Beijing. The Beijing isolates underwent the same change pattern reported from Guangdong Province. The same series of mutations occurring in separate geographic locations and at different times suggests a dominant process of viral adaptation to the host.

## Abstract 2

Viral adaptation to the host may be occurring under selective immune pressure.

Severe acute respiratory syndrome (SARS) is a new infectious disease that spread worldwide in early 2003, affecting >30 countries, with >8,098 cases and 774 deaths reported (*1*). Beijing, People's Republic of China, experienced the largest SARS outbreak in the world, with 2,523 cases and 181 deaths by June 12, 2003 (*2**,**3*). The epidemic occurred in 2 phases. The first phase began on March 5, 2003, and was caused by a patient who had been infected in Guangzhou and was involved in a superspreader event (SSE) in Beijing hospitals. Most patients in this period proved to be directly or indirectly linked with the index patient by traditional epidemiologic investigations. Molecular epidemiology, based on genome sequencing of the early isolates, also provided evidence that Beijing infections were closely related to those from the Guangdong epidemic (*4*). The second phase was marked by widespread transmission in healthcare facilities and communities, with incidence peaking in late April, followed by a dramatic decline in occurrence during the first week of May. The last probable case was noted on May 29, 2003 (*5*). During this phase, many case-patients had no apparent contact with SARS patients.

After the sequencing of the whole genome (*6**–**9*) information on viral strains from different geographic and temporal origins became available in GenBank. Comparative sequence analyses identified 5 loci, sequence variants of which segregated together as specific genotypic patterns, which could be used to define epidemic phases (*10*). All or some of the 5 loci were included in previous molecular epidemiologic studies (*4**,**11**–**13*), making them important genetic signatures to differentiate lineage-specific and temporal-specific patterns. In this study, we investigated the genetic variations of SARS-CoV in Beijing based on the 5-locus signature. Also, by sequence comparison among patients from 1 case cluster and different samples from 1 patient, the adaptable mutation of the virus in the host was further explored.

## Methods

### Participants

Study participants were recruited from 2 hospitals designated for SARS patients in Beijing. All of them fit the World Health Organization (WHO) case definition for probable SARS, i.e., temperature >38°C, cough or shortness of breath, new pulmonary infiltrates on chest radiograph, and a history of exposure to a SARS patient or of living in an area of on-going SARS transmission (*14*). After informed consent was obtained, epidemiologic and clinical data were collected from the participants by using a standard data collection form with interview and medical record review. The information obtained included the following items: age, sex, occupation, medical history, time and nature of exposure, symptoms and physical findings, laboratory tests at admission to hospital, and outcomes on discharge or transfer. Patients also provided clinical specimens (sputum and stool) for SARS-CoV detection by RT-PCR assay with specific primers (COR1, COR2) recommended by WHO. Only the patients with positive RT-PCR results were included in the study.

### Laboratory Methods

Specimens were analyzed by using RT-PCR techniques. Briefly, total RNA was extracted by using the QIAamp virus RNA mini kit (Qiagen, Hilden, Germany) as instructed by the manufacturer. RNA was used to synthesize cDNA with the SuperScript II RNase H^–^ reverse transcriptase system (Invitrogen, Carlsbad, CA, USA). Five sets of primers were used in nested PCR to amplify the fragments covering the 5-locus genetic signatures (17564, 21721, 22222, 23823, and 27827) (Table 1). Then, with the purified PCR products as templates and the second round primers as sequencing primers, the fragments were sequenced in ABI Prism 377 DNA sequencer (Applied Biosystems Inc, Foster City, CA, USA). Each PCR fragment was directly sequenced from both inward and outward directions, in duplicate.

**Table 1:** Primers used for nested polymerase chain reaction and sequencing

Position	Amplification region*	Primer sets (starting from 5´)	
17564	17440–18281	Forward	ACGTCTATATTGGCGATCCT TGTGCAGACTTATGAAAACAATA
		Reverse	GTTTTGCATTAACTCTGGTG GTTAGTACCCACAGCATCTCTAGT
21721	21585–22304	Forward	GATGATGTTCAAGCTCCTAATTAC CTTAACAGAGCATTTGAGTTCAG
		Reverse	CAACATACTTCATCTATGAGGGG TGTACCATTTTCATCATACTTGAG
22222	22177–22874	Forward	AGATGTAGTTCGTGATCTACCTTC TTAATGGCCAATAACAATTAAGA
		Reverse	CAAATTTTAGAGCCATTCTTACAG GGAGAAAGGCACATTAGATATGTC
23823	23455–24263	Forward	CGACACTTCTTATGAGTGCG ATGCAGTTGATGTTGTTGTAAG
		Reverse	GCATTTGTGCTAGTTACCATACAG TGATGTTGTTGTAAGTGATTCTTG
27827	27449–28270	Forward	CCATCAGGAACATACGAGG GACCACTATTGGTGTTGATTG
		Reverse	TAGCACACACTTTGCTTTTG CAGTATTATTGGGTAAACCTTGG

*The nucleotide position was given with TOR2 as the reference strain (accession no. NC004718).

All the original base data were processed for base calling, assembly, and editing by the SegMan II sequences analysis software of DNA Star package (DNASTAR, Madison, WI, USA). The comparisons with other sequences available from public database (GenBank) were made by using the default parameter of ClustalW (http://www.ebi.ac.uk/clustalw/). Single nucleotide variations (SNVs) were indicated, and the deduced amino acid changes were described.

## Results

A total of 160 samples (81 stools and 79 sputum samples) from 62 patients with positive results by RT-PCR were included this study. Of these, 45 samples (36 sputum samples and 9 stools) from 29 patients (17 men and 12 women, with a median age of 32 years) yielded amplicons for the 5 targeted loci (Table 2). The patients came from 2 SARS-designated hospitals in Beijing, with disease onset ranging from March to May, 2003. Four patients had serious conditions during hospitalization, including pulmonary aggravation requiring oxygen ventilation or transfer to an intensive care unit. No patient died.

**Table 2:** Epidemiologic and phylogenetic data on 29 severe acute respiratory syndrome patients, Beijing, 2003*

Patient no. (sex, age [y])	Onset date†	Sampling date,† clinical sample	5-loci genotype	Other variant loci
1‡ (M, 25)	3/10	4/28, Sp	GGCTC	22589
2‡§ (F, 48)	3/21	4/28, Sp	GGCTC	22589, 27749
3 (M, 19)	3/31	4/28, Sp; 5/5, Sp	GGCTC	22589
4‡ (F, 34)	3/31	5/5, St; 4/28, Sp	TGTTT	17620, 22589
5‡§ (M, 21)	3/31	4/28, Sp; 5/5, Sp	TGTTT	22589
6‡ (F, 34)	4/2	4/28, Sp	TGTTT	22077
7 (F, 27)	4/2	4/28, Sp	GGCTC	22589
8‡ (M, 31)	4/3	4/28, Sp	TGTTT	22077, 22589, 27749
9‡ (M, 20)	4/5	5/5, Sp; 4/28, Sp	TGTTT	17620, 22077, 22589
10 (F, 23)	4/8	5/22, St; 5/15, Sp	GGCTC	22589
11§ (M, 47)	4/8	5/5-Sp; 5/5, St	GGCTC	22589
12 (M, 73)	4/9	4/28, Sp; 4/28, St	TGTTT	22589, 27749
13 (M, 54)	4/9	4/28, Sp; 4/28, St	GGCTC	22589, 27749
14‡ (F, 21)	4/11	4/28, Sp; 4/28, St; 5/5, Sp	TGTTT	
15§ (M, 61)	4/12	5/22, Sp	GATTC	22589
16‡ (F, 25)	4/15	5/5, Sp	TGTTT	17620, 22589, 27749
17 (M, 25)	4/17	5/5, Sp	TGTTT	22077, 22589, 27749
18 (F, 20)	4/18	4/28, Sp; 4/28, St	TGTTT	22589, 27749
19‡ (F, 25)	4/20	5/5, Sp; 5/5, St	TGTTT	22077, 22589,
20 (F, 34)	4/21	4/28, Sp; 5/5, Sp	TGTTT	22077
21 (M, 33)	4/23	5/5, Sp; 5/5 St; 4/28-Sp	TGTTT	17620, 22589
22 (M, 28)	4/24	5/5, Sp	TGTTT	22589, 27749
23 (M, 61)	5/1	5/15, Sp	GATTC	22589
24‡ (F, 31)	5/1	5/5, Sp	TGTTT	22077, 2589, 7749
25 (M, 25)	5/2	5/7, Sp	TGTTT	22077
26‡ (M, 25)	5/4	5/22, Sp	TGTTT	22077
27 (M, 19)	5/6	5/22, Sp	TGTTT	22589, 27749
28 (F, 28)	5/7	5/22, Sp	TGTTT	17620, 22589, 27749
29 (M, 22)	5/12	4/28, Sp; 5/5, Sp	TGTTT	22589, 27749

*F, female; M, male; Sp, sputum; St, stool. †All dates are in 2003. ‡Patients were from the same cluster. §Patients with adverse clinical outcome.

The sequences of the 45 positive specimens were compared with SARS-CoV genome sequences available from the public database (GenBank). The sequence variants in 5 loci (17564, 21721, 22222, 23823, and 27827) defined 3 kinds of motifs: GGCTC, TGTTT, and GATTC (Table 2). In addition, 4 new SNVs were identified at nucleotides 17620, 22077, 22589, and 27749 in >1 patient. These variations appeared independently in several isolates, which indicates that they are not RT-PCR artifacts. None of them had been previously reported, with 3 nucleotide substitutions leading to amino acid changes (Table 3).

**Table 3:** Characterization of nucleotide (nt) substitutions in 29 severe acute respiratory syndrome patients, Beijing, China*

ORF or protein	Position†	nt substitution	aa change	No. patients
ORF 1b	17620	C→T	Leu→Ser	5
S protein	22077	G→T	Phe→Tyr	9
S protein	22589	C→T	Noncoding region	24
ORF 9	27749	G→A	Lys→Glu	12

*SARS, severe acute respiratory syndrome; ORF, open reading frame; aa, amino acid. †The nt positions are numbered with TOR2 as reference strain (accession no. NC_004718).

Twelve patients in this study belonged to a cluster. They derived from an SSE indirectly linked with the earliest SARS patients in Beijing. The first 2 patients of this cluster, who became ill on March 10 and 21, respectively, harbored the GGCTC motif. The remaining patients, who became ill from March 31 to May 4, showed the TGTTT motif. Among patients outside of the cluster, 5 of 6 patients with onset date before April had the GGCTC motif, while the TGTTT motif became predominant later (9 of 11 patients until May 12). A new motif, GATTC, was found in 2 patients outside the cluster. In addition, no intrapatient variation was observed in the 5 amplicons from specimens collected at different times or from different sources (sputum or stools).

The possible role of genetic mutations in patients' prognosis was also investigated. The presence of nucleotide substitution was compared between 2 groups of patients: 1 with good prognosis (absence of pulmonary aggravation; n = 25) and 1 with adverse outcome (pulmonary aggravation 8–12 days after onset of symptoms requiring oxygen ventilation or transfer to ICU; n = 4). No mutation was found associated with disease severity (Table 2).

## Discussion

During the 2003 SARS epidemic, conventional epidemiologic investigation, aided by viral sequencing analysis, identified viral genetic signatures that are linked to geographic and temporal clusters of infection (*4**,**10**–**12**,**15**–**18*). Findings of these studies are summarized in the Figure, connecting the worldwide epidemic to a transmission event in hotel M in Hong Kong in late February 2003.

**Figure:**
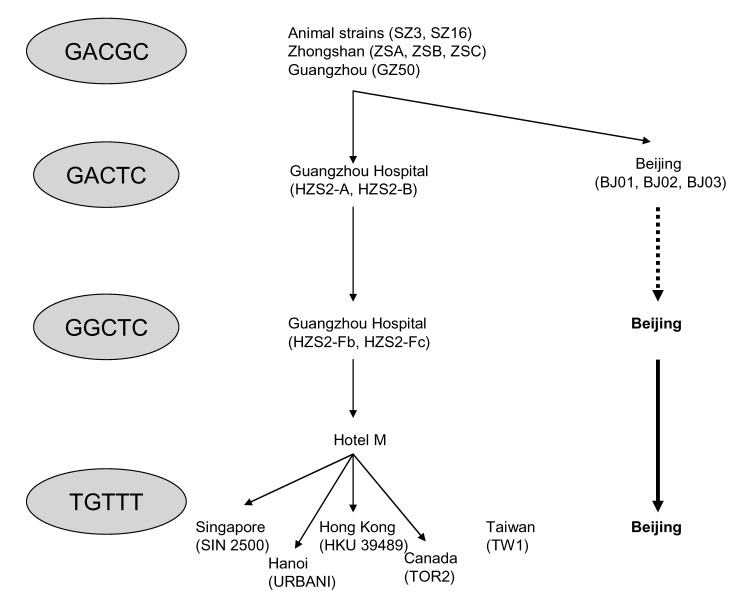
pidemiologic and phylogenetic links between patients of different worldwide SARS outbreaks (*4**,**10**,**11**,**12*). New information that concerns the Beijing epidemic is represented in **boldface**. Epidemiologic links that are still speculative are in dotted lines.

Beijing had experienced the SARS epidemic from March to June; however, only a few Beijing strains from the early epidemic have been analyzed in previous studies. Our study is the first to provide phylogenetic information on Beijing strains from the early and middle epidemic, as well as the late epidemic, by using the 5-locus motif of previous studies. The series of mutations in the 5-locus motif observed in Beijing followed the same path as isolates in Guangdong Province and the worldwide epidemic, i.e., the early introduction of GACTC motif was followed by transition to a GGCTC motif, before switching to a stable TGTTT motif. The observation of the same series of mutations occurring in 2 separate locations at different times suggests a dominant process of viral adaptation to the host. Moreover, this finding can expand our understanding of SARS-CoV response to selection pressures in humans, since early Beijing isolates (BJ01, BJ02, and BJ 03), which are traceable to Guangdong, underwent an independent selection process and would not be subject to the same sampling bias caused by superspreading events in Hong Kong isolates. The GGCTC→TGTTT switch was observed among patients belonging to the same cluster in this study, which rules out the possibility of the coincidental superposition of 2 epidemics (GGCTC and TGTTT) coexisting in Beijing.

The mutations involved in the GGCTC→TGTTT switch are responsible for amino acid changes in a nonstructural protein (17564, region Orf1b) in S protein (21721 and 22222) and in a noncoding region (27827, X3). We were not able to identify a correlation between these changes and the clinical status of patients. We did not find sequence variations in specimens obtained from the same patients either collected at different times or among different specimen types, which suggests that within-individual variations are rare in the partial genome of this study, although the phenomenon was described in a previous study (*15*). A new motif, GATTC, which represents a new transitional motif between GACTC and TGTTT, was described on 2 occasions in patients who were not part of the cluster. Similarly, 4 new SNVs were identified at nucleotides 17620, 22077, 22589, 27749.

In summary, this study confirms the evolution of SARS-CoV strains towards a TGTTT motif in positions 17564, 21721, 22222, 23823, and 27827 in Beijing, as was observed in Guangdong province before the hotel M outbreak in Hong Kong. Whether this motif is associated with higher transmission or virulence remains to be elucidated.
